# Behavioral Stress Fails to Accelerate the Onset and Progression of Plaque Pathology in the Brain of a Mouse Model of Alzheimer's Disease

**DOI:** 10.1371/journal.pone.0053480

**Published:** 2013-01-11

**Authors:** Qiuju Yuan, Huanxing Su, Wing Hin Chau, Cheung Toa Ng, Jian-Dong Huang, Wutian Wu, Zhi-Xiu Lin

**Affiliations:** 1 School of Chinese Medicine, Faculty of Science, The Chinese University of Hong Kong, Hong Kong, China; 2 State Key Laboratory of Quality Research in Chinese Medicine and Institute of Chinese Medical Sciences, University of Macau, Macao, China; 3 Department of Biochemistry, Li Ka Shing Faculty of Medicine, The University of Hong Kong, Pokfulam, Hong Kong, China; 4 Department of Anatomy, Li Ka Shing Faculty of Medicine, The University of Hong Kong, Pokfulam, Hong Kong, China; 5 State Key Laboratory of Brain and Cognitive Sciences, Li Ka Shing Faculty of Medicine, The University of Hong Kong, Pokfulam, Hong Kong, China; 6 Research Center of Reproduction, Development and Growth, Li Ka Shing Faculty of Medicine, The University of Hong Kong, Pokfulam, Hong Kong, China; 7 Institute of Central Nervous System Regeneration, Jinan University, Guangzhou, China; “Mario Negri” Institute for Pharmacological Research, Italy

## Abstract

Conflicting findings exist regarding the link between environmental factors and development of Alzheimer's disease (AD) in a variety of transgenic mouse models of AD. In the present study, we investigated the effect of behavioral stress on the onset and progression of Aβ pathology in the brains of TgCRND8 mice, a transgenic mouse model of AD. One group of TgCRND8 mice was subjected to restraint stress starting at 1 month of age until they were 3 months old, while restraint stress in the second group started at 4 months of age until they were 6 months old. After 2 months of treatment, no differences in the soluble, formic acid extracted, or histologically detected Aβ deposition in the cortical and hippocampal levels were found between non-stressed and stressed mice. These results showed that restraint stress alone failed to aggravate amyloid pathology when initiated either before or after the age of amyloid plaque deposition in TgCRND8 mice, suggesting that if stress aggravated AD phenotype, it may not be via an amyloid-related mechanism in the TgCRND8 mice. These findings are indicative that plaque load per se may not be used as a significant criterion for evaluating the effect of stress on AD patients.

## Introduction

Alzheimer's disease (AD), the most prevalent form of senile dementia, is characterized by two major histopathological hallmarks including Aβ plaque and tau-laden neurofibrillary tangle formation [1]. Although several genetic factors are known to be involved in early onset of familial AD [2]–[6], the etiology of sporadic AD that accounts for the majority of AD cases remains unclear [7]; [8]. Epidemiological studies suggest that AD can be modulated by environmental factors. For example, those who are prone to psychological distress are more likely to develop AD [9]; [10]. Although it is well accepted that both genetic and environmental factors are likely to trigger the pathogenic pathways of AD, researchers over the last decade have mainly focused on studying the genetic contributions in AD [11]–[13]. Studies have recently begun to investigate the effect of environmental factors on neuropathology and cognitive function in transgenic models of AD [13]–[16]. In contrast to the clinical observations that environmental factors play important roles in the complex etiology of AD [17], contradicting findings from animal models of AD have been reported. For example, environmental enrichment, such as increased physical activity, cognitive stimulation, or a combination of both, has been demonstrated to elicit different outcomes including a reduction [18]–[21], no effect [14]; [22]; [23], or even an exacerbation [24]; [25] in extracellular plaque pathology in animal models of AD.

Similar to environment enrichment, stress is another important paradigm that researchers often used to study the association of environmental factors and AD pathology in AD models. Stress, an unavoidable condition of human experience including both major life events and the problems of daily life, is known to affect the body's physiology [26]; [27], immunological response [28] and endocrine system [29]. The most popular experimental procedure to induce stress in animals relies on the use of restraint [30], which has the advantage of being straightforward and painless [30].

The experiments which subjected the mice of AD models to behavioral stress also yielded inconsistent results in terms of extracellular plaque pathology. For example, Devi et al [16] found that stress aggravated β-amyloidogenesis in hippocampus but not cortex, and in female but not male mice. In contrast, Lee et al [31] reported that the stress accelerates β-amyloidogenesis in not only cortex and hippocampus but also both female and male animals. Thus, the association of stress and β-amyloidogenesis remains an unresolved issue and clearly warrants further investigations.

The TgCRND8 mouse model has been demonstrated to develop a very early and aggressive phenotype, showing onset of Aβ pathology at the age of 3 months [32]. The aim of the present study was to determine whether restraint stress was able to accelerate the onset and progression of Aβ pathology in this mouse model by using animals of 1 (before Aβ plaque formation) and 4 month-old of age (after Aβ plaque formation) [32]. In the previous studies involved in investigating the effects of restraint stress on neuropathology of AD, common to all methods of the restraint is the restriction and immobilization of movement. However, a number of variations in effecting the restraint have been published. For example, the treatment duration varies ranging from a consecutive several days [16] to several months [33]. No comparative studies of the relative merits favoring any duration have been reported. In this study, we investigated the effects of two months of immobilization on the Aβ plaque formation in TgCRND8 mice.

## Materials and Methods

### Transgenic mice

The generation of TgCRND8 mice has been described previously [32]. TgCRND8 mice express a transgene incorporating both the Indiana mutation (V717F) and the Swedish mutations (K670N/M671L) in the human amyloid-beta protein precursor (APP) gene. The mice were kept on a C57BL6/J genetic background. Because many studies indicated that when stressed, male rodents showed habituation while female ones showed sensitization [16]; [34]; [35], only female mice were used in the current study. This study was carried out in strict accordance with the recommendations in Code of Practice for Care and Use of Animals for Experimental Purposes of Hong Kong. Procedures of animal handling were approved by the Committee on the Use of Live Animals for Teaching and Research of the University of Hong Kong. For sacrificing animals, sodium pentobarbital anesthesia was used to minimize suffering.

### Stress induction

TgCRND8 mice at 1- or 4 month-old were randomly assigned to either standard housing or restraint stress condition (n = 5). Standard laboratory cages (33 cm×18 cm×14 cm) were used for standard housing. The restraint treatment was performed as described previously [31]. Briefly, TgCRND8 mice were individually placed in a well-ventilated plastic tube. Mice were not able to move forward or backward while in the tube. The mice were restrained for 6 h per day. After each stress session, the mice were returned to their normal home environment, in which they were housed in standard laboratory cages with free access to food and water. This daily procedure was repeated for a consecutive 2 months.

### Thioflavin S Staining

Cross sections of the brains of the animals were incubated in 0.5% thioflavin S (Sigma-Aldrich, St Louis, MO, USA) in 50% ethanol for 10 min, differentiated twice in 50% ethanol, and washed in PBS solution. Staining was visualized under a Zeiss fluorescence microscope (Gottingen, Germany).

### Immunohistochemistry

For immunofluorescent staining, cross sections of the brain were incubated overnight at room temperature with primary antibodies (Bam-10, 1∶3000, Sigma-Aldrich, c-fos, 1∶3000, Chemicon and oxytocin, 1∶2000, Penisular laboratories). After rinsing with 0.01 M PBS, they were incubated for 1 h at room temperature with corresponding secondary antibodies conjugated with Alexa-488 or 568 (1∶800, Molecular Probes, Eugene, USA). The primary and secondary antibodies were diluted in PBS containing 1% normal goat serum and 0.2% Triton X-100. The number of c-fos-immunoreactive nuclei per section was counted in 5 sections per animal at the level of middle of supraoptic nuclei (SON).

For double staining of c-fos/oxytocin, the sections were first incubated with two primary antibodies derived from different species and then the corresponding Alexa 488 or 568-conjugated secondary antibody (1∶800, Molecular probes, Eugene, USA). Each step was followed by three washes in PBS. Finally, the sections on gelatin-coated glass slides were coverslipped in mounting medium (Dako, Denmark). Fluorescent images were captured with a Zeiss microscope (Gottingen, Germany) equipped with a Spot digital camera (Diagnostic Instruments, Sterling Heights, MI, USA).

For light microscopic inspection, spinal cord cross sections were rinsed three times for 10 min each in PBS. The sections were then immersed for 30 min in 0.2% H_2_O_2_ to inhibit endogenous peroxidase activity and blocked for 2 h with 5% normal serum in PBS/0.3% Triton X-100 and incubated with mouse Bam-10 overnight at room temperature. On the second day, the sections were incubated with secondary antibodies at room temperature. The sections were processed by the avidin-biotin-peroxidase/3,3-diaminobenzidine (DAB) method [36]. Each step was followed by three washes in PBS. The sections were then mounted on gelatin-coated slides for light microscopic inspection.

### Determination of Aβ plaque burden

Brains were coronally-sectioned in 30 µm thickness using a microtome. Plaque deposition levels were examined in paricortex and hippocampus. Five animals were used in each group for counting. To avoid bias in quantification of plaque levels, serial images of 100× magnification were captured using a Zeiss microscope equipped with a SPOT camera and SPOT software (RT Color diagnostic Instrument INC, Michigan, USA) on 6 sections per animal which were 30 µm afar from each other, starting 1.32 mm from bregma [14]. By using ImageJ software, pictures were binarized to 8-bit black and white pictures and a fixed intensity threshold was applied to define the DAB staining. Measurements were performed for a percentage area covered by Bam-10 DAB staining without knowing non-stress or stress treatment.

### Measurement of plasma corticosterone

Animals were anaesthetized by using katemine (80 mg/kg) and xylazine (8 mg/kg). Blood was collected from the heart within 3 min using a heparinized needle. Samples were centrifuged (2000 rpm for 20 min at 4°C). Plasma aliquots were stored at −80°C until use. The corticosterone level was examined by using a CorrelateEIA corticosterone kit (Assay Design, USA). Measurements were performed according to the manufacturer's instructions.

### Assessment of Aβ levels

Sandwich Aβ ELISA assay was performed as described previously [37]. Hippocampal tissue was homogenized in Tris-buffered saline (20 mM Tris and 137 mM NaCl, pH 7.6) supplemented with protease inhibitors. The formic acid-soluble Aβ was collected, and neutralized with 1 M Tris buffer (pH 11). The levels of Aβ1–40 and Aβ1–42 peptides were analyzed using human β Amyloid Aβ1–40 and Aβ1–42 Aβ colorimetric sandwich ELISA kits (Wako Pure Chemical Industries Ltd., Japan) according to the manufacturer's instructions.

### Statistical analysis

All data were given as means ± standard error of the mean (SEM). Data distribution was evaluated and student *t* test was then used to test the difference between non-stressed and stressed groups. A value of *p*<0.05 was considered statistically significant.

## Results

### Age-related Aβ deposition in the brain of TgCRND8 mice

**Figure 1:**
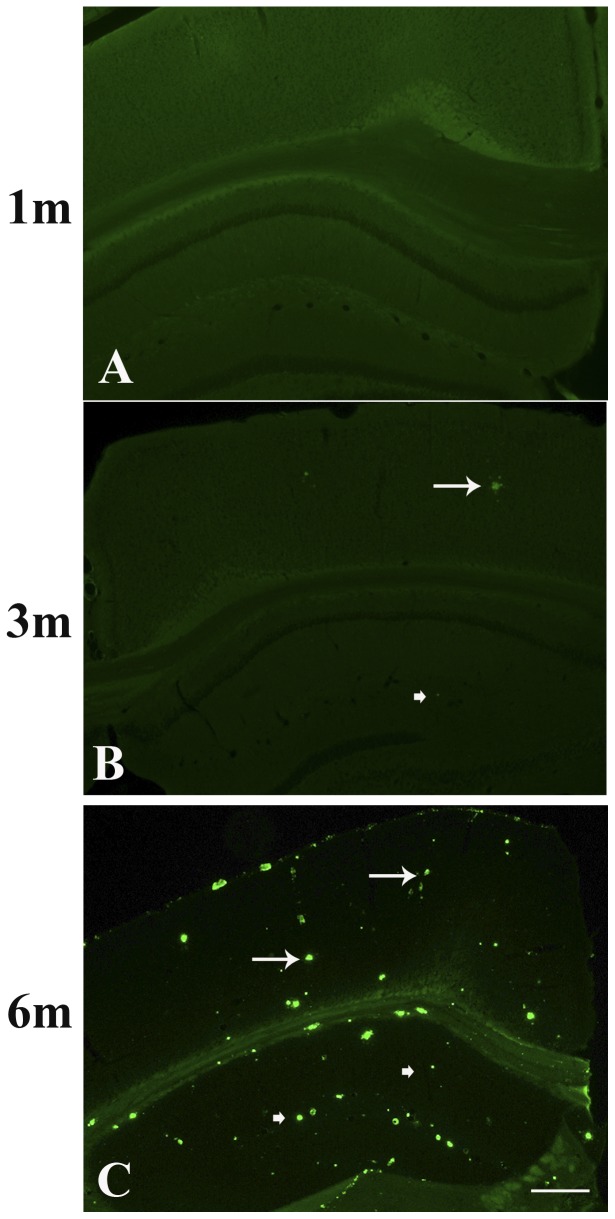
Cross sections of the brains were stained with Thioflavin S staining in TgCRND8 mice at the age of 1 (A), 3 (B) and 6 (C) months. Scale bar = 100 µm.

Consistent with previous studies [32], no Aβ plaque was found in either cortex or hippocampus of TgCRND8 at the age of 1 month (Fig. 1A, Table 1), and Aβ plaques could be observable in either cortex (arrow Fig. 1B, Table 1) or hippocampus (arrow head in Fig. 1B, Table 1) at the age of 3 months. It was found that Aβ plaques increased with age and abundant plaques were observed in cortex (arrows in Fig. 1C, Table 1) or hippocampus at the age of 6 months (arrow heads in Fig. 1C, Table 1).

**Table 1:** The number of Thioflavin S-positive plaques/section in the brains of TgCRND8 mice at the age of 1, 3 or 6 month-old.

Age of animals	Number of plaques in the brain/section
1 month-old	0
3 month-old	19±4.5
6 month-old	212±29

### Restraint stress activated hypothalamic neurons in TgCRND8 mice

**Figure 2:**
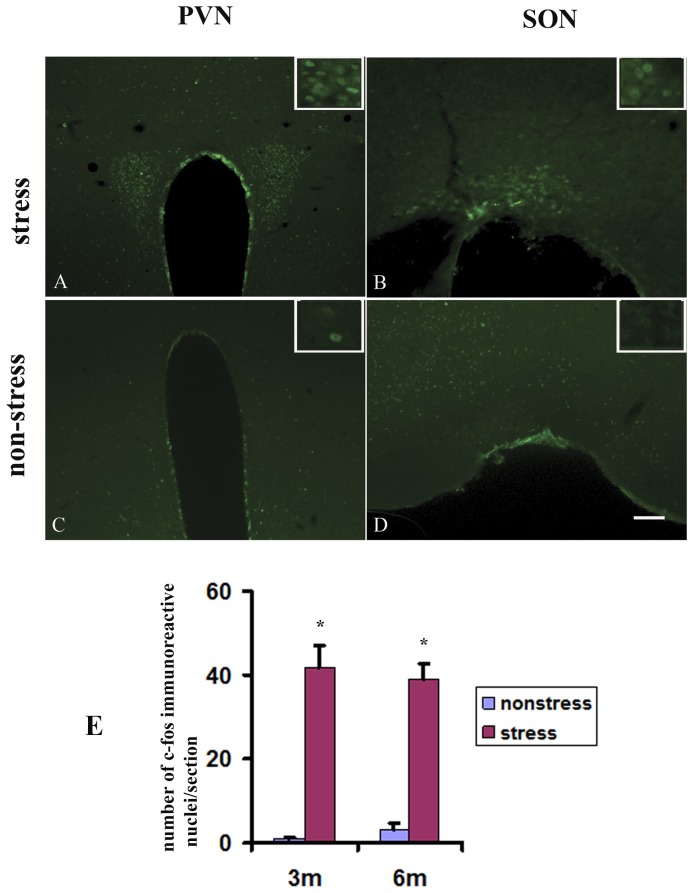
Restraint stress activated hypothalamic neurons in TgCRND8 mice. A–D: Cross sections of the brains stained with c-fos immunohistochemical staining in PVN (A and C) and SON (B and D) of TgCRND8 mice at the age of 4 months undergone restraint stress (A and B) and non-stress treatment (C and D). E: Quantitative analysis of number of c-fos immunoreactive nuclei in SON of stressed and non-stressed TgCRND8 mice. * indicates statistical differences when compared with their age-matched non-stressed controls at *p*<0.01. Scale bar = 150 µm.

**Figure 3:**
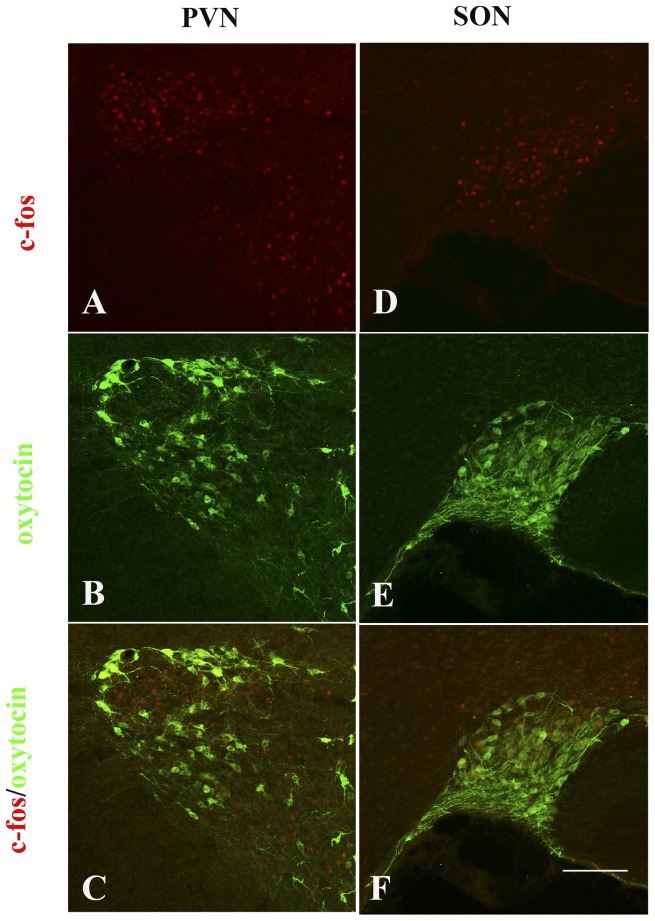
c-fos was induced in oxytotic neurons. A–C: Double staining of c-fos (red)/oxytocin (green) in paraventricular (PVN). D–F: Double staining of c-fos (red)/oxytocin (green) in supraoptic nuclei (SON). Scale bar = 75 µm.

To determine whether the restraint treatment induces stress response on TgCRND8 mice, we examined whether the neurons in hypothalamus was activated by restraint stress treatment. Consistent with previous study [38]–[40], few c-fos, if any, could be observed in both paraventricular (PVN) (Fig. 2C) and SON (Fig. 2D and E) of TgCRND8 mice. However, stress induced intensive c-fos expression in the two nuclei (Fig. 2A and B, respectively). Quantitative analysis also showed a significant difference in the number of c-fos immunoreactive nuclei in SON between the stressed and non-stressed animals (Fig. 2E). Co-labeling of c-Fos and oxytocin in PVN (Fig. 3A–C) and SON (Fig. 3E–F) further confirmed that c-fos was induced in the oxytocin-neurons of hypothalamus. The findings corroborated that restraint stress was able to induce stress response in hypothalamus of the experimental mice.

### Restraint stress increased the plasma corticosterone level of TgCRND8 mice

**Figure 4:**
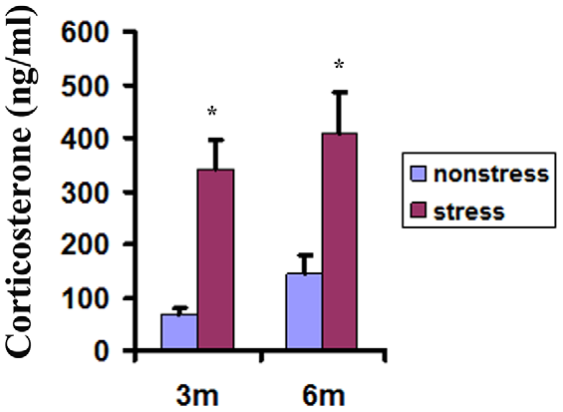
Plasma corticosterone levels in the stressed TgCRND8 mice increased compared with that in their non-stressed controls at the age of 3 and 6 month-old. * indicates statistical differences when compared with their age-matched non-stressed controls at *p*<0.01.

The stress response by the restraint treatment was further confirmed by the increased plasma corticosterone level of TgCRND8 mice. The basal levels of circulating corticosterone in TgCRND8 mice were 69.2±12.5 ng/ml at the age of 3 month-old and 146.5±32.1 ng/ml at the age of 6 month-old (Fig. 4). After the 6 h-2 m stress, the plasma corticosterone levels in TgCRND8 mice significantly increased to 341.1±57.3 and 409±76.3 ng/ml, respectively (Fig. 4).

### Restraint stress did not influence cortical and hippocampal amyloid plaque loads

**Figure 5:**
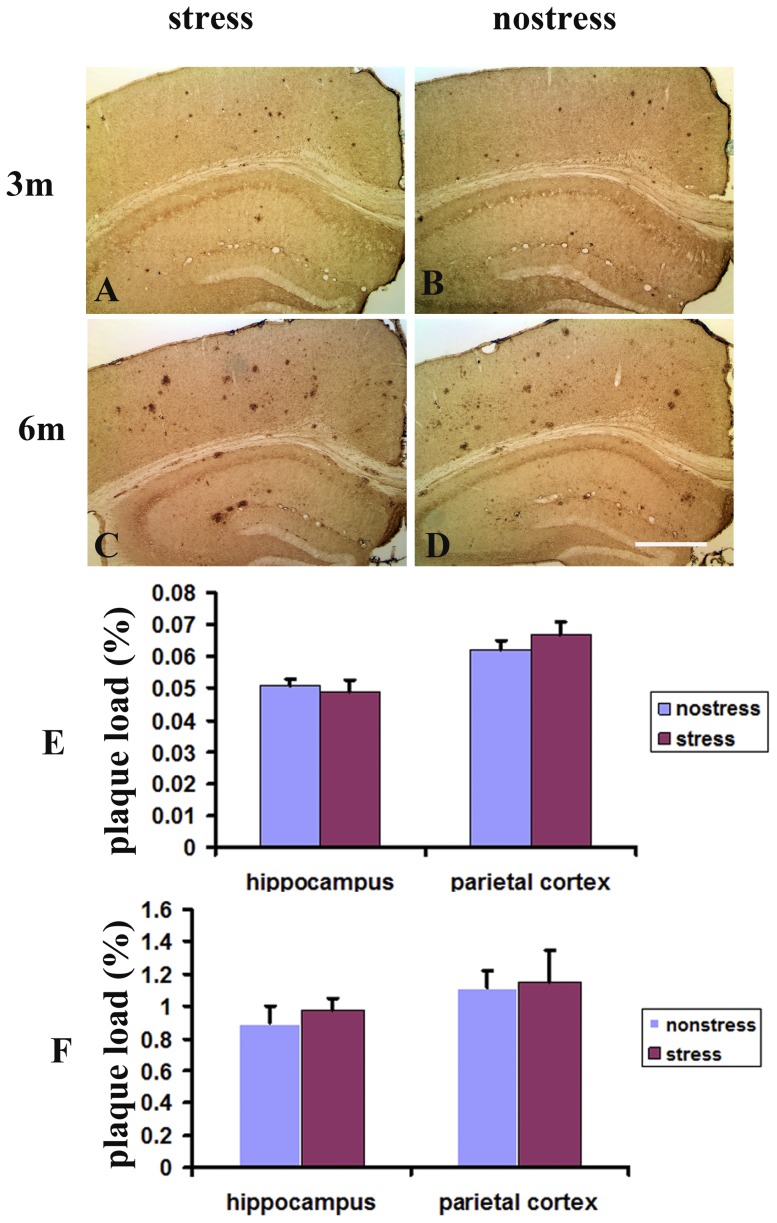
Restraint stress did not influence cortical and hippocampal amyloid plaque loads. A–D, Cross sections of the brains stained with bam-10 immunohistochemical staining in TgCRND8 mice at the age of 3 (A, B) or 6 months (C, D) under stress (A, C) and non-stress (B, D). E–F, Quantitative analysis of Aβ deposit burden in either cortex or hippocampus in TgCRND8 mice at the age of 3 (E) or 6 months (F) under stress or non-stress. Scale bar = 300 µm.

**Figure 6:**
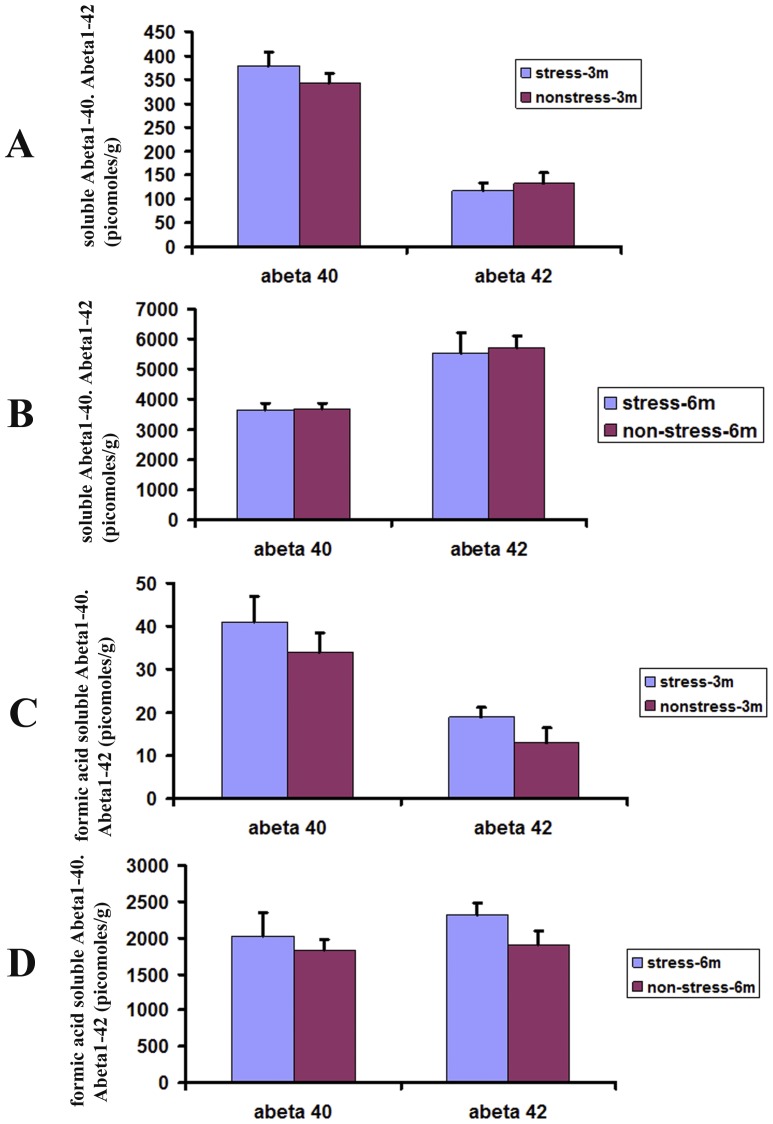
Effects of restraint stress on Aβ1-40 or Aβ1-42 levels in TgCRND8 mice. ELISA was used to measure Aβ levels in hippocampal tissues after completion of the restraint stress procedure. The data were expressed as means ± SEM. Restraint stress had no significant effect on Aβ levels in either soluble fraction or nonsoluble fraction.

To test whether behavioral stress directly affects the onset and progression of Aβ pathology, TgCRND8 mice at the age of 1 or 4 months were subjected to restraint stress treatment for 6 h daily for 2 months. Histopathologic analysis indicated that sparse Aβ plaques were detected in the brains of TgCRND8 mice at the age of 3 months (Fig. 5B). However, the stress did not increase the number of plaques in either cortex or hippocampus of the brains of the stressed animals (Fig. 5A). High plaque-load was found in the brains of the animals at 6 months of stressed mice (Fig. 5C), but the number of Aβ plaques in either cortex or hippocampus of stressed mice (Fig. 5C) did not exceed that in the non-stressed mice (Fig. 5D). Quantitative analysis also showed no significant difference in plaque load in either cortex or hippocampus in TgCRND8 mice at the ages of 3 (Fig. 5E) and 6 (Fig. 5F) months between the stressed and non-stressed animals.

### Restraint stress did not affect Aβ levels in hippocampus

The findings of unchanged Bam10-positive Aβ deposits were further corroborated by Aβ ELISA analysis in hippocampus of the brains. Soluble Aβ was first extracted with lysis buffer (Sigma-Aldrich, Poole, UK) (Fig. 6A and C), and the remaining Aβ was pelleted at 100,000 g and extracted with 70% formic acid (Fig. 6B and D). In either lysis buffer or formic acid extractable fraction, neither Aβ1–40 nor Aβ1–42 was found to be increased in the restraint stress mice when compared to their non-stressed controls.

## Discussion

Despite an intensive activation of the neurons of hypothalamic PVN and SON and marked increased levels of corticosterone, the stress marker, under restraint stress, this treatment paradigm failed categorically to modify Aβ pathology in the brains of TgCRND8 mice with treatment being initiated at the age of either 1 or 4 months. In this study, we applied 1- and 4-month-old TgCRND8 animals since the animals at the age of 1 month were not old enough to have amyloid plaque pathology in cortex and hippocampus, whereas the animals at the age of 4 month had an observable amyloid plaque pathology in their brains. These results indicate that the restraint stress failed to accelerate not only the onset of amyloid plaque pathology, but also its progression. These results were in stark contrast to those of Lee et al [31] who found the aggravation of Aβ pathology in restraint-treated Tg2576 mice despite both their and our groups used the same method to restrict the movement of animals. Both groups placed the animals in a plastic tube instead of other methods, e.g. taping the limbs of animals to a board or tie them to pads. Differences between the previous reports and the present study include: 1) intensity and duration of restraint, and 2) mouse line of AD model. Indeed, under certain circumstances, restraint will not produce a stress response which may be due to insufficient intensity and duration of the restraint [30]. In the study of Lee et al [31], AD mice were exposed to restraint for 2 h daily for consecutive 16 days. However, the animals in the present study were subjected to restraints for 6 h daily for 2 months, and the intensity of treatment was stronger and duration of treatment is much longer than those in the previous study. Thus, difference in intensity and duration of restraint seemed not to be the reason for the observed difference. To further confirm that the restraint produced a stress response in the restrained mice, we examined the global consequence of restraint stress in hypothalamus of neuroendocrine system [38]–[42], and found that restraint stress induced activation of oxytocin neurons in PVN and SON of hypothalamus as evidenced by induction of c-fos expression. It has been suggested that oxytocin may regulate stress-induced corticotropin-releasing hormone gene expression [38].

The different genotypes of the AD models may account for the different results seen in the studies of Lee et al and ours. In line with this, discrepancies in the reported results about studying the effects of environmental enrichment on Aβ pathology among different research groups have been reported. For example, on the one hand, environmental enrichment attenuates Aβ plaques in TgCRND8 mice [19], on the other hand, environmental enrichment exacerbates amyloid plaque formation in APP/PS1 transgenic mice [24]. More surprisingly, Cotel and co-workers [14] have recently found that environmental enrichment has no effect on Aβ load in APP/PS1KI mice. The conflicting findings might be attributed, at least partially, to the use of different transgenic models of AD. Our finding together with the those of Lee et al [31] may confirm the hypothesis that interactions between environmental risk factors and genetic background may influence the onset and progression of sporadic AD [15].

It has also been shown that effect of behavioral stress on beta-amyloidogenesis is sex-specific [16]. Devi et al [16] reported that behavioral stress increased plaque burden in the hippocampus of female 5×FAD mice but not in the male 5×FAD mice. However, the difference between our findings and those of Devi et al cannot be attributed to the difference in gender as we only used TgCRND8 female mice to evaluate the effect of restraint stress on Aβ pathology.

Our present study which showed the restraint stress failed to accelerate the onset and progression of Aβ pathology in TgCRND8 does not necessarily mean that our results are conflict with the clinical observations that stress may be an important contributor to the onset and development of AD [9]; [10]. Indeed, amyloid is only one part of a multi-factorial disease processes of AD incorporating a wealth of disease-causing factors [43]. Under certain circumstances, factors that either aggravate or attenuate AD-like pathology may not act via Aβ-related mechanisms. For example, Jeong et al [44] have found that in their stress system, memory function was impaired under stress even though Aβ deposition was unchanged. Similarly, Jankowsky et al [25] have shown that environment enrichment has a protective effect on cognitive function regardless of exacerbation in Aβ deposition. Our results provide evidence to support the notion that plaque load per se may not represent a significant outcome measure for evaluating the therapeutic effect on AD patients.

It is worth mentioning that the psychological and physiological changes associated with restraint appear to result from the distress and aversive nature of having to remain immobile, rather than immobilization itself [30]. In our study, we observed that restrained animals kept struggling even in a restrainer with a very limited space (See Video S1). The struggling of the mice increased, in a sense, physical activity. If so, this might explain why the restraint stress failed to aggravate the Aβ pathology in TgCRND8 mice, for it has been shown that higher levels of physical activity can attenuate Aβ pathology [20]; [21]. The physical activity might counteract the effect of the psychological stress on Aβ pathology in the present study. The evidence that increased physical activity as well as enriched housing can reverse the effect of stress on the progression of AD in a mouse model of AD also lend support to the above explanation [44]. The implication of our findings is that even if we constantly experience various types of stress (in fact, we are invariably and unavoidably confronted with stressful circumstances throughout life), some forms of effective interventions, e.g, increasing physical activity, can always be applied to confront and offset the adverse effects of stress on the onset and progression of AD pathology.

## Supporting Information

Video S1 **The restrained TgCRND8 mice are struggling in the plastic tube restrainer.**
(WMV)
